# Highly discordant serology against *Trypanosoma cruzi* in central Veracruz, Mexico: role of the antigen used for diagnostic

**DOI:** 10.1186/s13071-015-1072-2

**Published:** 2015-09-17

**Authors:** Daniel Guzmán-Gómez, Aracely López-Monteon, María de la Soledad Lagunes-Castro, Carolina Álvarez-Martínez, Manuel Jesús Hernández-Lutzon, Eric Dumonteil, Angel Ramos-Ligonio

**Affiliations:** Doctorado en Ciencias Biomédicas, Universidad Veracruzana, Xalapa, Veracruz Mexico; LADISER Inmunología y Biología Molecular, Facultad de Ciencias Químicas, Universidad Veracruzana, Orizaba, Veracruz Mexico; Centro de Investigaciones Biomédicas, Universidad Veracruzana, Xalapa, Veracruz Mexico; Laboratorio de Parasitología, Centro de Investigaciones Regionales “Dr. Hideyo Noguchi”, Universidad Autónoma de Yucatán, Mérida, Yucatán, Mexico; Department of Tropical Medicine, Tulane University, School of Public Health and Tropical Medicine, New Orleans, LA USA

**Keywords:** Chagas disease, Serodiagnostic, ELISA, Antigen, Antibodies

## Abstract

**Background:**

Chagas disease is a parasitic disease caused by the protozoan parasite *Trypanosoma cruzi.* In Mexico, the burden of the disease is difficult to estimate and improving surveillance for Chagas disease is an important priority. We aimed here at determining the seroprevalence of *T. cruzi* infection in humans in a rural community in Veracruz.

**Methods:**

Serum samples (196) were analyzed for *T. cruzi* infection using five enzyme-linked immunosorbent assay (ELISA) tests: two in-house tests based on crude parasite extract and three commercial ELISA kits. Because of highly discordant results, we further explored the importance of parasite antigens and strains by western-blot analysis.

**Results:**

A total of 74 samples (37.7 %) were reactive with at least one ELISA, but discordance among tests was very high. The best agreement was between Chagatest recombinant and Chagatek ELISA (Kappa index = 0.798). The agreement between other combinations of tests ranged from 0.038 to 0.518. Discordant samples were confirmed by western-blot analysis using up to nine parasite strains, giving a seroprevalence of 33.7 %.

**Conclusions:**

Commercial tests had a very limited ability to detect *T. cruzi* infection in the study population. In-house tests based on crude parasite antigens showed a greater sensitivity but were still unable to detect all cases of *T. cruzi* infection, even when based on a local parasite strain. The high seroprevalence confirmed the hyper-endemicity of *T. cruzi* infection in the region. Reliable epidemiological surveillance of Chagas disease will require the development of improved diagnostic tests.

**Electronic supplementary material:**

The online version of this article (doi:10.1186/s13071-015-1072-2) contains supplementary material, which is available to authorized users.

## Background

Chagas disease is a parasitic disease caused by the protozoan parasite *Trypanosoma cruzi* and transmitted primarily by hematophagous triatomine bugs. The disease is a major public health problem, affecting 8–10 million persons worldwide for a disease burden of over 29 million disability-adjusted life-years (DALYs), and a global health care cost reaching $627 billion [1, 2]. The disease in endemic in the Americas, where vector species are widespread [3], but it is also becoming globalized due to international migrations [4]. In Mexico, estimates indicate that there are at least 1–2 million infected persons, but possibly up to 6 million, although *T. cruzi* infection remains highly underreported [5, 6] and the true burden of the disease is difficult to estimate. Updating and improving surveillance and data for Chagas disease in Mexico should thus be an important priority, and help improve access to treatment for Mexican patients [7].

Serologic diagnostic for *T. cruzi* infection has undergone great progress in recent years, with the development and commercialization of many assays based on different parasite antigens. Indeed, there has been a shift in recent years from whole parasite extracts to more defined recombinant antigen mixtures showing better specificity [8–10]. The reported sensitivity and specificity of most of these assays appears very high (often >98 % for both). Nonetheless, their actual performance has been found to be somewhat lower [11] and no gold-standard has been identified for an accurate and reliable diagnostic of *T. cruzi* infection. As a consequence, the World Health Organization and most National guidelines in Latin American countries still recommend the use of two tests based on different principles and antigens, and in case of discordance, a third test can provide a final diagnostic.

Differences in test performance have often been attributed to the parasite stage used for antigen preparation, with amastigotes, epimastigotes and trypomastigotes potentially having a different antigenic profiles [12, 13]. Also, tests based on whole parasite lysate or crude antigens are believed to be much less reproducible and more difficult to standardize than those based on recombinant antigens [8–10]. The diversity of parasite strains has also been incriminated, leading to the recommendation that antigens from local strains should be favored to ensure optimum sensitivity and specificity of the tests. Indeed, *T. cruzi* has been divided into at least six discrete typing units (DTUs), referred to as TcI-TcVI, with very distinct characteristics [14]. Nonetheless, we previously found a good agreement between commercial tests, including a rapid test and a recombinant ELISA test, and an in-house ELISA based on a crude extract from a local parasite strain from Mexico [15], suggesting that all these tests may be useful for the serodiagnostic of *T. cruzi* infection in Mexico [16]. ELISA tests based on a variety of TcI strains from Mexico and Guatemala and the CL-Brener TcVI strain were found to perform similarly, suggesting that these strains were equally good sources of antigens for the detection of *T. cruzi* infection [17]. Indeed, agreement among tests is relatively high in most studies [18, 19], although the combination of tests implies that a small proportion of the samples will present discordant results, which are complicated to manage from a epidemiological and clinical point of view [19, 20]. Also, a low sensitivity of tests currently used can be a potential cause of underdiagnosis [11].

In this study, we aimed at determining the seroprevalence of *T. cruzi* infection in humans in a rural community in central Veracruz, using a combination of five ELISA tests based on different antigenic preparations. Due to highly discordant results, we further explored the possible role of parasite antigens and strains used in the serologic diagnostic of *T. cruzi* infection.

## Methods

### Serum samples

The study was carried out in the rural community of Las Josefinas, in central Veracruz, Mexico (−96°41′31″ and 18°28′26″). Information on Chagas disease and on the project was provided to the inhabitants of the community during open meetings organized in the rural medical unit of the community. Interested participants were given an appointment for themselves and their family to provide blood samples. The day of the appointment, written informed consent was obtained from each volunteer, and blood samples were collected in vacutainer tubes. Serum was separated by centrifugation at 1,200 × g for 10 min and samples were stored at −70 °C until used. A total of 196 serum samples were collected and analyzed for *T. cruzi* infection using five different tests: two in-house enzyme-linked immunosorbent assay (ELISA) based on crude parasite extract, three commercial ELISA diagnostic kits, and a western blot analysis.

### In-house ELISA

Two in-house ELISA tests were used, based on epimastigote crude extracts of the CL-Brener (belonging to TcVI DTU) and LJ01 strains (TDIM/MEX/2014/LJ01/T. cruzi, a mixture of TcI and non-TcI DTUs, isolated from *Triatoma dimidiata* collected in the village of Las Josefinas, Veracruz, Mexico), respectively. Both strains were cultured in liver infusion tryptose medium supplemented with 10 % (w/v) fetal calf serum. Briefly, logarithmic phase parasites were harvested by centrifugation at 1,000 × g for 10 min at 4 °C. The parasite pellet was suspended in 500 μL of phosphate-buffered saline (PBS) (137 mM NaCl, 2.7 mM KCl, 4.3 mM Na_2_HPO_4_, and 1.4 mM KH_2_PO_4_, pH 7.4) and lysed by cycles of freezing (−70 °C) and thawing (25 °C). The suspension was centrifuged at 10,000 × g for 20 min at 4 °C. The resulting supernatant (extract) was used as crude antigen extract. Protein concentration was determined by the Bradford method. In-house ELISA protocol was performed as follows, polystyrene plates (Costar Corporation, Cambridge, MA) were coated with the *T. cruzi* crude antigen extract (10 μg/mL) in carbonate buffer, pH 9.6, and incubated overnight at 4 °C. Unbound antigen was removed and plates were blocked with 200 μL of PBS containing 5 % weight/volume (w/v) non-fat milk for 2 h at 37 °C. After washing with buffer (PBS with 0.05 % w/v Tween 20), the plates were incubated with 50 μL of serum samples (1:200 dilution with PBS); each sample was assayed in duplicate, and plates also included positive and negative control serum samples. Further washing steps were performed and a peroxidase labeled goat anti-human IgG antibody (Pierce, Rockford, IL) was added at a 1:5,000 dilution in PBS with 0.05 % w/v Tween 20 and incubated for 1 h at room temperature. After eight washes, 100 μL of 2,2,-azino-bis (3-ethylbenzthiazoline)-6-sulphonic acid (Zymed, San Francisco, CA) was added as substrate and the reaction was allowed to proceed for 20 min at room temperature. The reaction was stopped with 2 % w/v sulfuric acid, and absorbance was read at 450 nm with an ELISA microplate reader (Multiscan MS; Labsystems, Vantaa, Finland).

### Commercial ELISA diagnostic tests

We use three different commercial ELISA diagnostic kits, Chagatest ELISA recombinant v3.0 from Wiener lab (Rosario, Argentina) is based on six recombinant proteins (1, 2, 13, 30, 36 and SAPA antigens), reported to be conserved among strains. The manufacturer reports a sensitivity of 99.3-100 % and a specificity of 98.7–100 %, and an agreement with other reference methods of 99.6 %. Chagatek ELISA from Lemos/Biomerieux (Santiago del Estero, Argentina) is based on purified *T. cruzi* antigens, and has a reported sensitivity of 100 % and a specificity of >99 %. The NovaLisa® Chagas (*Trypanosoma cruzi*) IgG ELISA from NovaTec Inmunodiagnostica Gmbh (Dietzenbach, Germany) has a reported sensitivity and specificity of >99 %, and is based on *T. cruzi* recombinant antigens. Assays were performed and cut-off determined according to the respective manufacturer’s instructions.

### Western blot analysis

Western blot was carried out as described before [21] using a panel of nine different *T. cruzi* strains from three DTUs. Briefly, 25 μg of crude antigen extract from the MHOM/BR/1978/Sylvio-X10 (TcI DTU), CIET1, Camp8, Nayarit and H1 (all Tc I), MHOM/BR/1950/Y (TcII), TINF/CL/1945/Tulahuen (TcII), and TINF/BR/1963/CL-Brener (TcVI) strains were separated by electrophoresis in a 10 % sodium dodecyl sulfate–polyacrylamide gel, 4 replicates were performed and electroblotted onto a nitrocellulose membrane (Bio-Rad, Hercules, CA) at 80 volts at 4 °C for 1 h. The membranes were blocked with 5 % w/v solution of nonfat milk powder and washed with TBST buffer (50 mM Tris–HCl, pH 7.4, 150 mM NaCl, 0.05 % Tween 20). The nitrocellulose membrane was individually incubated (2 h at 37 °C) with 1 ml of different human sera diluted 1:100 in TBST with 2 % skim milk. Control membranes were incubated with positive and negative serum samples. Each membrane was washed three times with TBST and subsequently incubated with alkaline phosphatase–labeled goat anti-human IgG (Pierce). The membranes were then washed as above and the immune complexes were developed with nitrotetrazolium blue (NBT) and 5-bromo-4-chloro-3-indolyl phosphate (BCIP). The reaction was stopped with water. Positive, negative, and secondary antibody controls were included in each experiment.

### Statistical analysis

Cut-off OD values for commercial ELISA tests were determined as recommended by each manufacturer. For in house ELISA tests, the cut-off was defined as the mean OD of negative serum samples (*n =* 20) plus three standard deviations. Frequencies of reactive samples were calculated for each ELISA tests. The agreement between different serological tests was assessed using the Kappa index. Seroprevalence rates were calculated for subgroups of the study population according to sex and age, as well as their 95 % confidence intervals (CI). Differences among groups were assessed by *Χ*^2^ tests.

## Results

### ELISA assays

**Fig. 1:**
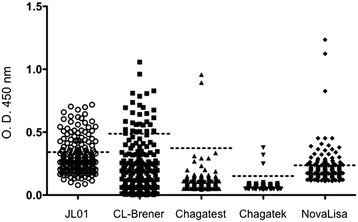
Reactivity of human sera with different ELISA tests. Five different ELISAs were used to identify positive samples to *T. cruzi*, two in-house ELISAs (JL01 and CL-Brener extracts), and three commercial kits. The dotted lines indicate the cut-off line for each ELISA test

A total of 196 blood samples were collected from inhabitants of the village of Las Josefinas, Veracruz, and tested with five ELISA tests for *T. cruzi* antibodies (Fig. 1). A total of 122 (62.2 %) samples were negative with all the tests, while 74 (37.7 %) were reactive with at least one test. However, discordance among tests was very high, with a total of at least 63/196 (32.1 %) samples with discordant ELISA results. Only one sample (0.5 %) was reactive with all five tests, and another one (0.5 %) with four of the five tests, and thus unambiguously considered as *T. cruzi* positives (Table 1). There were an additional nine samples (4.5 %) presenting reactivity in three ELISA tests, although with different combinations of tests. Eight samples (4 %) were reactive with NovaLisa® Chagas IgG test, and the two ELISA with total extracts from the CL-Brener and LJ01 strains (Table 1), and one sample (0.5 %) was reactive with Chagatek ELISA (Laboratorio Lemos/Biomerieux), NovaLisa® Chagas IgG (NovaTec Inmunodiagnostica), and the LJ01 total extract ELISA. A further 29 samples (14.8 %) were reactive with different combinations of two ELISA tests. Sixteen samples (8.1 %) were reactive with both in-house ELISA based on total *T. cruzi* extracts, but with none of the commercial tests, 12 samples (6.1 %) were reactive with NovaLisa® Chagas IgG test, and the total extract from the LJ01 strain, and one sample with NovaLisa® Chagas IgG test, and the total extract from the CL-Brener strain. Finally, an additional 34 samples (17.3 %) were reactive with a single ELISA test, with 23 samples (11.7 %) reactive with the in-house ELISA with the LJ01 strain, 9 samples (4.6 %) were reactive with the NovaLisa® Chagas IgG test, and another 2 samples (1.0 %) with the CL-Brener ELISA test only (Table 1).

**Table 1:** Reactivity of samples against different test ELISA

Group			Tests			Number of reactive samples (%)
	LJ01	CL-Brener	NovaLisa	Chagatek	Chagatest	
1	R	R	R	R	R	1 (0.5 %)
2	R	N	R	R	R	1 (0.5 %)
3	R	R	R	N	N	8 (4.0 %)
4	R	N	R	R	N	1 (0.5 %)
5	R	R	N	N	N	16 (8.1 %)
6	R	N	R	N	N	12 (6.1 %)
7	N	R	R	N	N	1 (0.5 %)
8	R	N	N	N	N	23 (11.7 %)
9	N	N	R	N	N	9 (4.6 %)
10	N	R	N	N	N	2 (1.0 %)
11	N	N	N	N	N	122 (62.2 %)
Total^a^	62 (31.6 %)	28 (14.3 %)	33 (16.8 %)	3 (1.5 %)	2 (1.0 %)	196 (100 %)

Note: The sum of the values and percentages (^a^) of the last row does not reflect the total number of samples, but refers to the samples which were reactive for each test

Groups 1–4: Samples reactive for more than three tests

Groups 5–7: Samples reactive for two tests

Groups 8–10: Samples reactive for one test

Group 11: Sample negative for all test

*R* Reactive, *N* Negative

Based on a minimum of two reactive tests, a total of 40 samples (20.4 %) could thus be considered positive. However, there was a very poor agreement among tests, as indicated by very low Kappa indexes (Table 2). Indeed, the best agreement was between Chagatest recombinant 3.0 and Chagatek ELISA, with a Kappa index reaching 0.798. The agreement between other combinations of tests was very low, with Kappa indexes ranging from 0.038 to 0.518. Also, it remained unclear whether samples with a single reactive ELISA should be considered as false positive or not.

**Table 2:** Kappa index among serological tests

	A	B	C	D	E
A	-	0.518	0.339	0.185	0.044
B		-	0.205	0.038	0.049
C			-	0.143	0.097
D				-	0.798
E					-

A: Crude extract LJ01 strain ELISA

B: Crude extract CL-Brener ELISA

C: NovaLisa® Chagas IgG (NovaTec Inmunodiagnostica)

D: Chagatek ELISA (Laboratorio Lemos/Biomerieux)

E: Chagatest Recombinant 3.0 (Wiener)

### Western blot assays

**Fig. 2:**
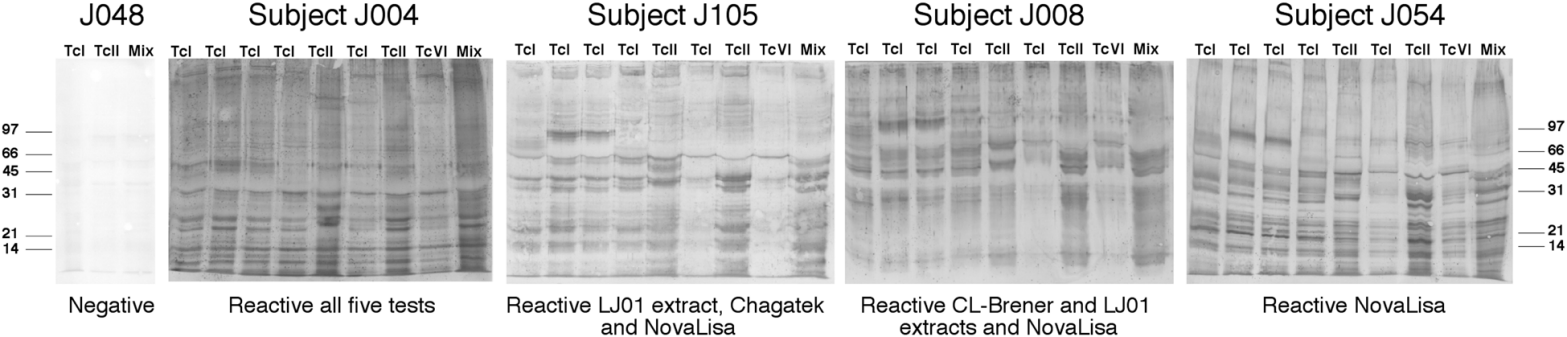
Western blot analysis of serum samples against different *T. cruzi* strains and DTUs. Serum samples were tested for their reactivity against a panel of eight *T. cruzi* crude antigen from different strains and DTUs. These include, from left to right: Silvio-X10 (TcI), Ciet-1 (TcI), Camp-8 (TcI), Nayarit (TcI), Y (TcII), H1 (TcI), Tulahuen (TcII), CL-Brener (TcVI), and LJ01 (Mixture of TcI and non-TcI)

Because discordant results from *T. cruzi* serological test are often attributed to antigenic differences among parasite strains/DTUs or among recombinant proteins, we tested several of the serum samples in western blot assays using a panel of *T. cruzi* crude antigens from several parasite strains and DTUs, including five TcI, two TcII and one TcVI strains. As expected, the serum from Subject J004, which was reactive with all five ELISA tests, showed a strong banding pattern indicating a reactivity to many *T. cruzi* proteins for this serum (Fig. 2). Importantly, while the banding pattern varied clearly among the *T. cruzi* strains, this sample was strongly reactive with all strains and DTUs tested. In addition, serum samples which presented reactivity with different combinations of ELISA tests (Subjects J105 and J008, Fig. 2) also resulted reactive with different band patterns against all strains tested. A serum sample that was reactive with a single ELISA test was similarly reactive against all *T. cruzi* strains tested (Subject J054, Fig. 2). In fact, the serum samples reactive with a single ELISA (9 reactive with NovaLisa® Chagas IgG test, 11 with LJ01 in house ELISA, and two with CL-Brener ELISA, respectively) and that were tested by western blot resulted all reactive against both LJ01 and Tulahuen parasite extracts (Additional file 1: Figure S1). This clearly indicated that these samples were not false positives of their respective ELISA tests but true positives for *T. cruzi* antibodies.

### Seroprevalence in the study population

Based on the combination of ELISA tests and western-blot confirmation, a total of 62/184 samples (33.7 %; 95%CI [27.3; 40.8]) could thus be confirmed as positive with at least two tests. Men presented a significantly higher seroprevalence than women (55 vs 32 %, *X*^2^ = 5.63, *P* = 0.017). Although seroprevalence tended to increase with age, this was not statistically significant (36.8 vs 40.8 % for children <12 years old and >12 years old subjects, *X*^2^ = 0.16, *P* = 0.68), indicating a very active recent transmission of *T. cruzi* in this population.

## Discussion

We evaluated here the seroprevalence of *T. cruzi* infection in humans in a rural community in Veracruz, using a combination of five ELISA tests based on different antigenic preparations. Unexpectedly, we found a very high level of discordance among the ELISA tests used, with very poor agreement among them. The seroprevalence varied from 1–1.5 % with two of the commercial ELISA up to 31.6 % with an in-house ELISA based on a crude parasite extract from a local *T. cruzi* strain. Up to 32 % of the samples presented discordant results among the ELISA tests and could thus be considered as inconclusive. This is contrary to most studies, which show a relatively good concordance among tests, and only a 1–2 % of inconclusive results [18, 19]. However, a study in Brazil also reported a high discordance among hemaglutination, immunofluorescence and ELISA tests [22].

The reactivity of samples by western blot confirmed that commercial ELISAs used in our study were very poorly sensitive for the detection of antibodies against *T. cruzi* in the study population. This is contrary to previous studies in Brazil, which showed that Chagatek ELISA had a good sensitivity and specificity [19, 23–25]. Similarly, Recombinant Chagastest ELISA also performed well in Argentina [18], Colombia [26], Brazil [19, 24, 25], and southern Mexico [15, 16]. We found here that the most sensitive commercial test was NovaLisa® Chagas IgG test, detecting a seroprevalence of up to 16.3 %, but this still represented only half of the total seropositive cases confirmed by western blot. This test has not been used extensively so far, but seemed to perform very well in Colombia [27]. In particular, it presented very limited cross-reaction (1/7 serum samples) using sera from Leishmaniasis patients [27].

The differences in performance among commercial tests may be due to the number and nature of the antigen used, and a larger mixture of antigens may be more reliable than a few recombinant antigens [18, 28]. This may also explain why crude parasite lysates, presenting a very large number of parasite antigens, can be more sensitive than recombinant antigens [29]. Indeed, the in-house ELISA tests based on the CL Brener and a local *T. cruzi* strain were able to detect the largest number of reactive serum samples, which were confirmed by western blot. However, an important drawback of such crude antigens is their insufficient specificity due to potential cross-reactivity of sera from leishmaniasis patients [29], which has motivated the development of test based on recombinant antigens. In our case, serum samples that were reactive with in house ELISAS are very unlikely to be from leishmaniasis patients. Indeed, although leishmaniasis is known to occur in the state of Veracruz [30], very few cases have been reported in the region where this study was performed. In addition, the Chagatek ELISA test was previously found to effectively react with serum from leishmaniasis patients (17/21 serum samples) [24], but gave very few reactive samples in our study population.

The low sensitivity of the commercial ELISA tests may also be due to a loss or masking of epitopes in the corresponding antigens due to glycosylation and other post-translational modifications. Indeed, carbohydrates epitopes have been found to be the target of a substantial part of host immune responses to other parasites [31, 32]. In the case of *T. cruzi*, a recombinant trypomastigote surface protein presented a low sensitivity for the serologic diagnostic of infection when compared to an indirect immunofluorescence assay [33], which may indeed be due to a lack of post-translational modifications of the protein produced in *Escherichia coli*. Glycosylation and hydroxylation have also been found to modulate the reactivity of *T. cruzi* glycosphingolipids fractions with human sera from Chagasic patients [34].

As previously, western blot was found to be a very good method for the confirmation of the reactivity of serum samples against *T. cruzi* antigens [35]. Importantly, it confirmed the reactivity of the serum samples against a wide variety of parasite strains, covering a large diversity of local DTUs, as well as strains from other geographic regions. Although the recognition pattern somewhat varied according to the strain used, all parasite extracts were clearly recognized by all serum samples tested. Nonetheless, western blot remains a test difficult to perform on large numbers of samples and for routine diagnostic/confirmation. In addition, only continuous epitopes are detected in western blots since proteins are denatured, while ELISAs rely on native proteins, which may result in a higher non-specific background. Tests based on *T. cruzi* excreted-secreted antigens (TESA) have also showed to be good for confirmation of inconclusive serologies [20, 24, 36], but also share the limitation of been difficult to apply to large number of samples.

Improvement in current commercial ELISA tests is thus strongly needed. However, even the in-house ELISA test based on a local strain was unable to detect 11 *T. cruzi* positive serum samples (5.6 %), which were detected with the CL-Brener crude antigen or the NovaLisa® Chagas IgG tests. This suggested that the LJ01 parasite extract still did not include a repertoire of antigenic proteins that covered the entire *T. cruzi* diversity in the region. While the genetic diversity of *T. cruzi* was thought to be limited in Mexico, with TcI as the predominant DTU [37], several recent studies have found that other DTUs are also present and with high frequencies in both Mexico (TcII, TcIII, TcIV and TcV) and the United States (TcII) [38–40]. Thus, the identification and characterization of new parasite-specific antigens and epitopes [41–44] covering a wider range of the parasite antigenic profile may lead to the development of more specific and sensitive ELISA tests. Indeed, greater attention to parasite diversity should be considered for diagnostic development, similarly to what has been suggested for drug development [45]. Multiplex serology based on panels of novel recombinant proteins may provide an optimum diagnostic tool [46, 47], but need to be adapted to a test format that is easy to perform for the processing of large number of samples. In addition, the use of recombinant proteins expressed in eukaryotic systems, and thus with post-translational modifications, may improve their diagnostic accuracy.

Finally, the seroprevalence of *T. cruzi* infection we detected in the study population is very high, reaching 33.7 %. This confirmed our previous observations in this area, obtained with different ELISA tests [48]. Also, the continued presence of active transmission is evidenced by a high seroprevalence in children < 12 years of age (36.8 %), also in agreement with a significant house infestation by *Triatoma dimidiata* and its frequent human feeding [49]. Thus, improved epidemiological surveillance and vector control interventions are urgently needed in the region.

## Conclusion

In conclusion, we observed in this study a very high level of discordance among the ELISA tests used, with very poor agreement among them. Particularly, the commercial tests had a very limited ability to detect *T. cruzi* infection in the study population. In-house tests based on crude parasite antigens showed a greater sensitivity but were still unable to detect all cases of *T. cruzi* infection, even when based on a local parasite strain. Western-blot allowed the confirmation of an overall seroprevalence of 33.7 %, confirming the hyper-endemicity of *T. cruzi* infection in this region and the need for extensive epidemiological surveillance for a better prevention and control of Chagas disease. Nonetheless, this will require the development of improved diagnostic tests for an accurate detection of cases.

## Additional file

Additional file 1: Figure S1. Western blot of serum samples against different *T. cruzi* strains. Serum samples reactive with a single ELISA test were confirmed by western blot using crude extract of the *T. cruzi* LJ01 (A) and Tulahuen (B) strains. These serum samples were reactive with the LJ01 ELISA (lanes 1–11), CL-Brener ELISA (lanes 12–13), and the NovaLisa® ELISA (lanes 14–22). C+: positive control serum, C-: negative control serum. (JPEG 70 kb)
