# Supplementary figures and images for: Highly discordant serology against Trypanosoma cruzi in central Veracruz, Mexico: role of the antigen used for diagnostic
Source: Parasit Vectors. 2015 Sep 17;8:466. doi: 10.1186/s13071-015-1072-2 (PMC4573690; doi:10.1186/s13071-015-1072-2)

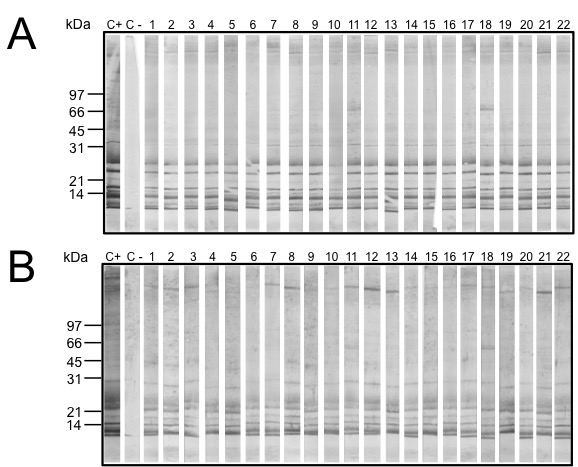

Supplement: Additional file 1: Figure S1. — Western blot of serum samples against different T. cruzi strains. Serum samples reactive with a single ELISA test were confirmed by western blot using crude extract of the T. cruzi LJ01 (A) and Tulahuen (B) strains. These serum samples were reactive with the LJ01 ELISA (lanes 1–11), CL-Brener ELISA (lanes 12–13), and the NovaLisa® ELISA (lanes 14–22). C+: positive control serum, C-: negative control serum. (JPEG 70 kb) [file 13071_2015_1072_MOESM1_ESM.jpg]
